# Identification of Clinically Relevant Protein Targets in Prostate Cancer with 2D-DIGE Coupled Mass Spectrometry and Systems Biology Network Platform

**DOI:** 10.1371/journal.pone.0016833

**Published:** 2011-02-11

**Authors:** Ramesh Ummanni, Frederike Mundt, Heike Pospisil, Simone Venz, Christian Scharf, Christine Barett, Maria Fälth, Jens Köllermann, Reinhard Walther, Thorsten Schlomm, Guido Sauter, Carsten Bokemeyer, Holger Sültmann, A. Schuppert, Tim H. Brümmendorf, Stefan Balabanov

**Affiliations:** 1 Department of Oncology, Haematology and Bone Marrow Transplantation with Section Pneumology, Hubertus Wald-Tumor Zentrum, University Hospital Eppendorf, Hamburg, Germany; 2 Bioinformatics, University of Applied Sciences Wildau, Wildau, Germany; 3 Department of Medical Biochemistry and Molecular Biology, University of Greifswald, Greifswald, Germany; 4 Interfacultary Institute of Genetics and Functional Genomics, University of Greifswald, Greifswald, Germany; 5 Department of Otorhinolaryngology, Head and Neck Surgery, University of Greifswald, Greifswald, Germany; 6 Cancer Genome Research, Deutsches Krebsforschungszentrum, Heidelberg, Germany; 7 Department of Pathology, University Hospital Eppendorf, Hamburg, Germany; 8 Prostate Cancer Center, University Hospital Eppendorf, Hamburg, Germany; 9 Aachen Institute for Advanced Study in Computational Engineering Science, RWTH Aachen University, Aachen, Germany; 10 Medizinische Klinik IV - Hämatologie und Onkologie, RWTH Aachen University, Aachen, Germany; Queensland University of Technology, Australia

## Abstract

Prostate cancer (PCa) is the most common type of cancer found in men and among the leading causes of cancer death in the western world. In the present study, we compared the individual protein expression patterns from histologically characterized PCa and the surrounding benign tissue obtained by manual micro dissection using highly sensitive two-dimensional differential gel electrophoresis (2D-DIGE) coupled with mass spectrometry. Proteomic data revealed 118 protein spots to be differentially expressed in cancer (n = 24) compared to benign (n = 21) prostate tissue. These spots were analysed by MALDI-TOF-MS/MS and 79 different proteins were identified. Using principal component analysis we could clearly separate tumor and normal tissue and two distinct tumor groups based on the protein expression pattern. By using a systems biology approach, we could map many of these proteins both into major pathways involved in PCa progression as well as into a group of potential diagnostic and/or prognostic markers. Due to complexity of the highly interconnected shortest pathway network, the functional sub networks revealed some of the potential candidate biomarker proteins for further validation. By using a systems biology approach, our study revealed novel proteins and molecular networks with altered expression in PCa. Further functional validation of individual proteins is ongoing and might provide new insights in PCa progression potentially leading to the design of novel diagnostic and therapeutic strategies.

## Introduction

Prostate cancer (PCa) is currently the leading cancer among men in western countries [1]. Autopsy studies have revealed that approximately 30% of men over the age of 50 years and 80% of men in their 70s have microscopic evidence of prostate cancer [2], [3]. In the year 2007 in the United States, an estimated 218,890 new cases diagnosed and 27,050 men died of PCa [4]. The (early) detection rate and thus the incidence of PCa has risen dramatically due to the introduction of PSA screening. Nonetheless, determination of serum PSA exhibit some major limitations, as elevated levels closely correlate with both hyperplasia and cancer.

Two-dimensional gel electrophoresis (2DE), a powerful tool used for protein separation and expression profiling is one of the core technologies in proteomics [5], [6]. Protein expression analysis of patient materials are informative led to the identifying cancer specific markers for diagnosis, therapeutic targets and is the basis for revealing various cellular events associated with cancer progression [7]. To date, several research groups have already performed protein and gene expression profiling studies on surgical and biopsy PCa specimens [8]–[12] but most of the potential reported markers are not in clinical application for definitive diagnosis of PCa. However, previous studies focussed on interindividual comparisons of proteomic analysis of radical prostatectomies from cancer patients to those of non cancer patients with conventional 2DE. Intraindividual analysis of tumor and benign tissue samples (adjacent to tumor) from the same cancer patients may reduce technical variability and thus provide a means to increase specificity of the results and to increase chances to identify specific disease-associated protein alterations.

In the present study, we investigated the comparative proteome of prostate cancer and its adjacent histological benign tissue from cancer patients with definitive pathological characterization in our 2-D differential in-gel electrophoresis (DIGE) system for protein 2-D electrophoresis [13] and MALDI-TOF-MS/MS for protein identification. The differentially expressed proteins were analysed by MetaCore™ (Gene GO) and Ingenuity Pathway Analysis program (Ingenuity Systems). The major hubs of significant sub networks were validated by real-time PCR analysis. Systems biology network analysis might provide the potential role of proteins in different regulatory pathways that control PCa progression and predict new biomarkers, which can be further validated using Western blotting and immunohistochemical (IHC) approaches.

## Materials and Methods

### Clinical Samples and Ethics Statement

Tissue samples and patient data were obtained with informed written consent. The study was approved by the Ethics Committee of the University Hospital Hamburg Eppendorf and carried out in accordance with the declaration of Helsinki. For protein expression analysis whole prostates were collected after radical prostatectomy from patients with elevated PSA values and preoperative pathological examination at Martini Clinics, Hamburg, Germany. Patients received no preoperative therapy. 24 patients were selected and the corresponding clinical and pathological data is provided are in Table 1. The serum PSA levels of these patients were determined and all patients had a range between 3.9 and 30.4 ng/ml (mean PSA value = 10.93 ng/ml) and a Gleason score between 3+3 and 4+5 [14], [15].

**Table 1:** List of patients included in the proteomic study together with their PSA levels, histology grading and tumor stage.

S. No	Patient No.	Tumor satge	Gleason Score	Pre operative hormone therapy	Pre operative PSA
1	T 4850	pT2c	3+4	NA	NA
2	T 4484	pT2c	3+4	No	8.6
3	T 2443	pT2c	3+3	No	7.9
4	T 2258	pT2a	3+3	No	3.9
5	T 3969	pT3b	4+3	No	30.4
6	T 3972	pT2c	3+4	No	13.77
7	T 2621	pT2c	3+4	No	7.53
8	T 2266	pT2a	3+4	No	5.53
9	T 3455	pT2c	3+3	No	5.5
10	T 2620	pT2c	4+3	No	9.51
11	T 4486	pT2c	3+4	No	29.41
12	T 2267	pT3a	3+4	No	NA
13	T 3974	pT3a	4+5	No	6.3
14	T 2437	pT2c	4+3	No	11.2
15	T 4167	pT3a	3+4	No	5.33
16	T 3132	pT2c	3+4	No	13.56
17	T 2442	pT2c	3+4	No	18.8
18	T 2933	pT2c	4+3	No	7.09
19	T 2259	pT2c	3+4	No	8.9
20	T 2936	pT2c	3+4	No	4.19
21	T 2434	pT2c	3+4	No	10.3
22	T 4766	NA	NA	NA	NA
23	T 3982	NA	NA	NA	NA
24	T 36126	NA	NA	NA	NA

After radical prostatectomy samples were frozen in liquid nitrogen until use. Tumor and benign areas were marked on the sections. We employed manual micro dissection method to obtain pathologically characterized materials for our proteomics approach. The corresponding areas on the remaining blocks were sliced out with sharp knife, embedded in Tissue-tek® and stored at −80°C until use.

### Protein isolation and labelling with CyDyes

Tissue was rinsed with physiological saline (0.9% NaCl) to remove residual mounting materials, marking dye and blood. The sliced tissues were directly homogenized in DIGE lysis buffer (30 mM Tris, 2 M Thio urea, 7 M Urea, 4% CHAPS; about 0.5 mL/200 mg tissues). The resulting homogenate was cleared to remove all debris by centrifugation at 12,000g for 15 min at 4°C, the protein supernatant was collected and its protein concentration was determined by a modified Bradford assay [16]. The quality of the samples for DIGE was evaluated by mini 2DE (7 cm IPG strips, pH 4–7). The protein lysates were labelled with Cy Dyes according to the manufacturer's protocol for minimal labeling (CyDye DIGE Fluor minimal dyes, GE Healthcare). In order to minimize dye-specific labeling artifacts, Cy3 and Cy5-labeling patterns were swapped among the same group of samples. An internal standard pool with equal amounts of each protein sample (25 µg) was used to reduce inter-gel variation. The pooled internal standards were labelled with Cy2. 50 µg protein of each sample was labelled with 400 *p*mol of corresponding dye on ice in the dark for 30 min. Reaction was quenched/stopped with 10 mM L-lysine for 10 min under the same conditions.

### DIGE-Two-dimensional gel electrophoresis (DIGE-2-DE)

The first dimension isoelectric focussing was carried out by using 24 cm immobilized pH gradient dry strips (IPG) with a linear pH 4–7 gradient. For analytical gels, a pair of Cy3 and Cy5 labelled samples (each 50 µg of protein) and 50 µg of Cy2 labelled internal standard were pooled and filled up to 150 µl with 2× sample buffer (7 M urea, 2 M thiourea, 4% CHAPS, 2% DTT) supplemented with 2% (v/v) IPG buffer pH 4–7). For rehydration, dilute samples with 2× rehydration buffer (8 M urea, 4% CHAPS, 13 mM DTT) supplemented with 1% (v/v) IPG buffer pH 4–7 and few grains of Bromophenol blue) to final volume of 450 µl. IPG strips (24 cm, pH 4–7, GE Health Care) were passively rehydrated overnight at 20°C in IPGPhor cassettes. For preparative gels, 750 µg of unlabelled protein pooled from equal amounts of samples was used. Proteins were separated by the PROTEAN IEF system (Bio-Rad) using a programmed voltage gradient at 20°C with a current limit of 50 µA per strip in the dark under the following conditions: 4 h at 250 V, 8000 V linear gradient to 15000 V hrs, rapid 8000 V to 75000 V hrs, for a total of 90 kVh. After IEF, the IPG strips were equilibrated in buffer 1 (50 mM Tris-HCl pH 8.8, 8 M urea, 30% glycerol, 2% SDS and 0.5% DTT) and buffer 2 containing 4.5% iodoacetamide instead of DTT in each case for 15 minutes.

Second dimension was performed in PROTEAN® Plus Dodeca™ Cell system. The equilibrated strips were applied to the top of 12.5% SDS-PAGE gels and sealed with 1% agarose prepared in SDS-Tris-glycine buffer with traces of bromophenol blue as a tracking dye to monitor electrophoresis. Polyacrylamide gels (12.5%) were cast in low fluorescence glass plates. Electrophoresis was performed with constant voltage (80V) at 20°C until the dye front reached the bottom of the gel. The complete apparatus is protected from light. Following electrophoresis, analytical gel cassettes were washed with ddH_2_O and wiped with dust free tissue paper. The Cy2 (internal standard), Cy3 and Cy5 labelled proteins in each gel were visualized by using a Typhoon 9400™ laser scanner (GE Healthcare) at 100 microns density by using different excitation and emission wavelengths directly from gels between glass plates. Optimal excitation/emission wavelengths for fluorescence detection are 488/520 nm for Cy2, 532/580 nm for Cy3, and 633/680 nm for Cy5. Preparative gels were stained with Roti®-Blue, a colloidal coomassie brilliant blue G250 stain. Briefly, gels were fixed in 40% methanol, 15% acetic acid for at least 4 hrs and then immersed in colloidal staining solution overnight. To remove background staining gels were washed in 20% methanol.

### Image analysis

Delta 2D differential analysis software version 4.0 (Decodon GmbH, Germany) was used in this study. For individual gel analysis, spots were detected, quantified and normalized according to the volume ratio of corresponding spots detected in the Cy2 image of the pooled-sample internal standard using the internal standard module. All normalized spot quantities from the gels were collectively analyzed as two independent groups “Tumor” and “Benign”, which enables matching of multiple gel images from different patients to provide statistical data on average abundance for each protein spot among the DIGE gels included in analysis. Three gels from benign group were excluded from analysis due to the problem in DIGE labelling. Student's *t*-test was performed to assess the statistical significance of differentially expressed proteins. Based on average spot volume ratio, spots whose relative expression is changed at least 1.5 fold (increase or decrease) between benign and tumors at 95% confidence level (t-test; p<0.05) were considered to be significant. For subsequent mass spectrometry analysis significant spot coordinates were transferred to coomassie stained preparative gel for spot picking.

### Mass spectrometry

#### Preparation of peptide mixtures for MALDI-TOF-MS/MS

Protein identification was performed as described recently [11]. Briefly, proteins were excised from Colloidal Coomassie Brilliant Blue stained 2-DE gels using a spot cutter. Digestion with trypsin and subsequent spotting of peptide solutions onto the MALDI-targets were performed automatically in the Ettan Spot Handling Workstation. Gel pieces were washed 50 mM ammoniumbicarbonate/50% (v/v) methanol and with 75% (v/v) ACN. After drying trypsin solution containing 20 ng/µl trypsin in 20 mM ammoniumbicarbonate was added and incubated at 37°C for 120 min. For peptide extraction, gel pieces were covered with 50% (v/v) ACN/0.1% (w/v) TFA and incubated for 30 min at 37°C. The peptide containing supernatant was transferred into a new micro plate and the extraction was repeated. The supernatants were pooled and dried completely at 40°C for 220 min. Peptides were dissolved in 0.5% (w/v) TFA/50% (v/v) ACN and spotted on the MALDI-target. Then, matrix solution (50% (v/v) ACN/0.5% (w/v) TFA) saturated with CHCA was added and mixed with the sample solution by aspirating the mixture five times. Prior to the measurement in the MALDI-TOF instrument, the samples were allowed to dry on the target 10 to 15 min.

#### MALDI-TOF-MS

The MALDI-TOF measurement of spotted peptide solutions was carried out on a 4800 MALDI TOF/TOF™ Analyzer. The spectra were recorded in reflector mode in a mass range from 800 to 4000 Da with an internal one-point-calibration on the autolytic fragment of trypsin (mono-isotopic (M+H)^+^ m/z at 2211.104, signal/noise ≥10). Additionally MALDI-TOF-MS/MS analysis was performed for the 5 strongest peaks of the TOF-spectrum after subtraction of peaks corresponding to background or trypsin fragments. The internal calibration was automatically performed as one-point-calibration if the mono-isotopic arginine (M+H)^+^ m/z at 175.119 or lysine (M+H)^+^ m/z at 147.107 reached a signal to noise ratio (S/N) of at least 5. After calibration a combined database search of MS and MS/MS measurements was performed using the GPS Explorer software v3.6 (Applied Biosystems, Foster City, USA). Peak lists were compared with the SwissProt rel.49 restricted to human taxonomy or IPI human v3.12 database using the Mascot search engine 1.9 (Matrix Science Ltd, London, UK). Peptide mixtures that yielded at least twice a mowse score of at least 56 for SwissProt or at least 59 for IPI database results were regarded as positive identifications.

### Bioinformatics analysis of the proteomic data

The significant differentially expressed proteins and their respective biological functions or relationships were determined using the KEGG database (http://www.genome.jp/kegg/) and Entrez protein database (http://www.ncbi.nlm.nih.gov/sites/entrez?db=protein) from NCBI. Protein networks for analyzing shortest pathways between the identified proteins were built by MetaCore™ (Gene GO) software and Ingenuity Pathways Analysis (IPA) program (Ingenuity Systems) for identifying molecular partners involved in particular disease. A master global network of all differentially expressed proteins (input objects) was created according to published literature-based annotations, and then the further sub networks were built from the master network to focus on activated experiments and/or pathways. Major hubs were identified based on the connections and edges with in the network. The protein expression data was analysed by hierarchical clustering and partition analysis with R-language to find potential markers which can classify all samples as tumor and benign with high certainty. The unsupervised clustering was performed using Euclidean distance measure and the agglomeration method ‘average’ of the log transformed values of all significant differentially expressed proteins of 45 samples included in the analysis set.

Lukk et al. showed that genome-wide differential expression between tumors and control tissue can be characterized using principal component analysis (PCA) by means of a malignancy parameter which characterizes coherent differential expression patters which are associated with tumor formation. In order to analyze the impact of co-regulation of protein expression on biomarker identification, unsupervised PCA has been applied on the protein expression data (normalized on logarithmic scale), both from tumor and normal tissue samples. The PCA was performed in Matlab [The Mathworks Inc, Version 14]. The resulting principal components of PCA are weighted means of the single protein expressions, where the weights are automatically identified such that the main variation of protein expression in the data set can be explained by the first few principal components.

In order to avoid spurious results from outliers, a two step approach was applied, where in a first step PCA was applied on the original data. The outlier detection was applied on the expressions of the principal components. In the second step the PCA was performed without the outliers. The resulting expression values for the stabilized principal components where used for further analysis. In order to analyze the ratio of information with respect to differential expression which is represented by the first three principal components and the residual space, for each protein the expression values for all tissues, quantified by the vector x_i_, where split into two components:
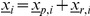
where x_p,i_ is the component of x_i_ which is represented by the first three principal components of the PCA (S3) and x_r,i_ quantifies the residual protein expressions in the complementary space CS3 which cannot be represented by the first three principal components of PCA. In order to check the distribution of differential expression between both components, for the original data given by x_i_, the PCA-based components x_p,i_, and the residual components x_r,i_ a two-sided t-test for differential expression between normal and tumor samples has been performed.

### RNA isolation and measurements of gene transcripts of interests by quantitative real time PCR

Quantitative real time PCR for analysis of transcriptional levels of proteins of interests was performed using SYBR Green as described previously. Briefly RNA was isolated from the same biopsies used for proteome analysis using TRIzol® reagent (Invitrogen, Karlsruhe, Germany) according to the manufacturers' protocol. The cDNA was prepared by reverse transcription of 1 µg total RNA using oligo dTprimer (15mer) and M-MLV reverse transcriptase (Fermentas Life Sciences GmbH, Germany). Quanti Tect primers for genes of interest GAPDH (housekeeping gene) were purchased directly from Qiagen, Germany. Primer sets were shown to generate a single amplicon of the desired size evaluated by RT PCR followed by agarose gel electrophoresis. Quantitative real time PCR was performed in thermal cycler (Stratagene, Germany) using Dynamo Flash SYBR Green qPCR kit (Finnzymes, Finland). PCRs for the target and housekeeping genes were performed in triplicates and mean relative expression levels were reported. Conditions for real time PCR reaction were as follows: 1 cycle of 94°C for 3 min and 40 cycles of 94°C for 20 s, 60°C for 30 s and 68°C for 30 s. At the end of the PCR, samples were subjected to a melting analysis to confirm specificity of the amplicon. To obtain statistical significance data obtained were analysed by unpaired student *t*-test performed and p value<0.05 was considered as significant.

### Western blotting

Protein extracts were separated by 12% SDS-PAGE and electrophoretically transferred onto PVDF membrane. Blocking was carried out in 1×Rotiblock solution (Roth Chemicals) followed by incubating the membrane with primary antibody overnight at 4°C. Excess antibodies were removed by washing with NaCl–Tris–Tween 20. Incubation with secondary antibody conjugated to horseradish peroxidase [anti-(mouse IgG) or anti-(rabbit IgG), diluted 1∶5000 in 1×Rotiblock] was performed for 1 h at room temperature. After three washes, the reaction was developed by the addition of LumiGLO substrate (Thermo). The emitted light was captured on X-ray film (GE Healthcare).

## Results

### 2D-DIGE Analysis and mass spectrometry

**Figure 1:**
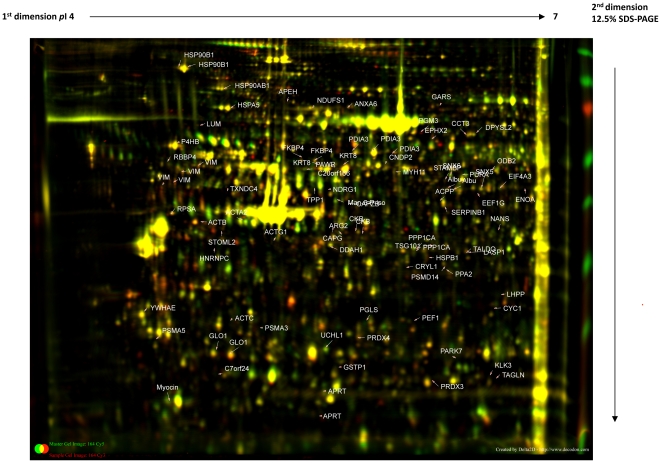
Representative 2-DE proteome map of prostate tissue from tumor vs. benign samples. Proteins were resolved by IEF over the pI range 4–7, followed by 12.5% SDS-PAGE and overlaid by Delta2D. After extraction from tissues, proteins were labeled with Cy3 and Cy5. An internal standard comprised of equal amount of proteins from all samples (benign and PCa groups) was labeled with Cy2 and included in all gels. The green spots indicate downregulated proteins, while the red spots indicate upregulated proteins in PCa relative to the corresponding benign tissue. The identified proteins that showed significantly altered expression in the PCa are indicated with arrows and labeled with the respectives protein IDs.

**Figure 2:**
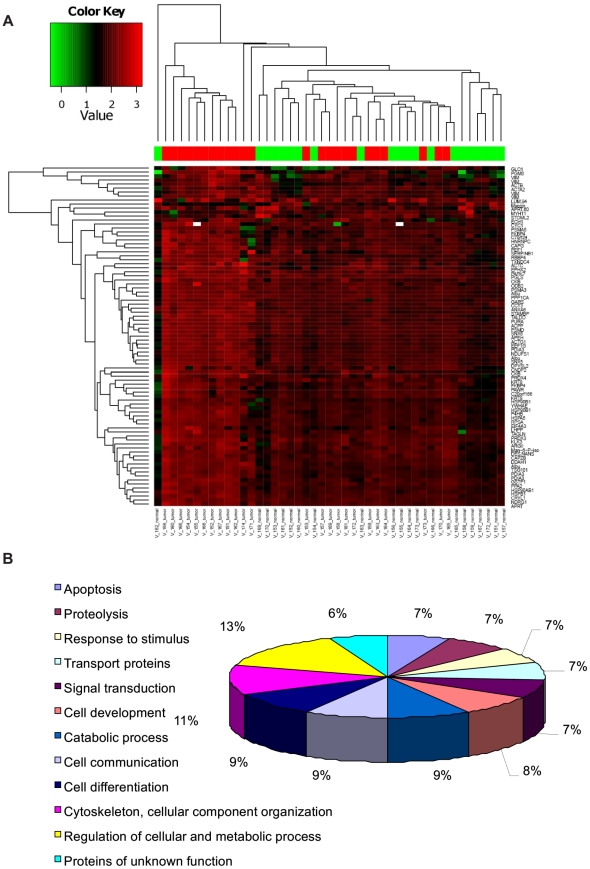
Cluster analysis and Gene Ontology of differential expressed proteins. (**A**) Unsupervised clustering (euclidean distance measure and the ‘average’ agglomeration method) was performed using the log transformed expression protein values of 45 samples. The samples are shown horizontally, the proteins vertically. The dendrograms represent the distances between the clusters. In the upper color bar, the tumor samples are marked in red, the normal tissues are shown in green. (**B**) Biological processes regulated by the all significant differentially expressed proteins assessed by Gene Ontology search and summarized according to their functions.

In this study, we were able to establish a standard procedure for manual micro dissection of radical prostatectomy samples to obtain pathologically evaluated tumor and benign tissues for proteome analysis. Furthermore, we analyzed the proteome of prostates from 24 cancer patients and their corresponding benign tissue in 21 cases (Table 1) by 2D-DIGE with the pI range of 4.0–7.0 and molecular weight range between 10 kDa and 120 kDa. Under these conditions, a total of 1324 spots were clearly detected and subsequently analysed using Delta2D software for differential protein expression. 118 spots were significantly altered in their abundance among all the samples included in the analysis set and were selected for further identification. The average abundances of spots were quantified and those with relative changes in abundance greater than 1.5 times between benign and tumor (up or down) at 95% confidence level (*p*<0.05) were considered as significant. Interesting spots were excised from preparative gels for protein identification (ID) by tryptic in-gel digestion and MALDI-TOF MS/MS analysis. Following a Mascot database search using the acquired MS data 96 spots of 79 proteins were identified as differentially expressed in cancer compared to benign tissue. The spots with protein ID are depicted in Figure 1. Individual proteins were reflected by multiple spots most likely due to posttranslational modification leading to shifts in the 2-DE. The proteins identified were grouped into different classes based on functional information available. Most of the identified proteins were either cytoskeletal proteins, enzymes of intermediary metabolisms, signal transduction, heat shock proteins, tumor-related proteins, oxidative stress related proteins or proteins of unknown function (Figure 2B). Details of the protein identifications, protein score, sequence coverage, theoretical pI value and molecular weight as well as average relative change are shown in Table S1.

### Hierarchical clustering and partition analysis of samples

The Figure 2A displays the clustering result. Higher expressions are coloured red, the lower ones in green. The samples are shown in columns and the rows indicate proteins. The dendrograms represent the distances between the clusters. The tumor and benign samples do not form two distinct separate clusters. However, hierarchical clustering revealed one group of very similar tumor samples (10 samples) form a cluster. These samples were considered as a tumor subgroup in further analysis. Partition analysis of the expression values resulted in the finding that a single protein, PPA2 can classify samples with significance (Fisher test with p-value 6.682e-09). The tumor subgroup was correctly classified, one (out of 14) of the remaining tumors was misclassified as benign and three (out of 21) of the benign samples were misclassified as tumors (data not shown). Partition analysis results have also shown many proteins can classify all samples correctly (Data not shown). In an attempt to assign a PSA specific protein signature, no direct correlation between PSA and differentially expressed proteins was observed (Data not shown). Taken together, more than one protein can distinguish all samples as they were assigned in the groups.

### Principal component analysis (PCA)

**Figure 3:**
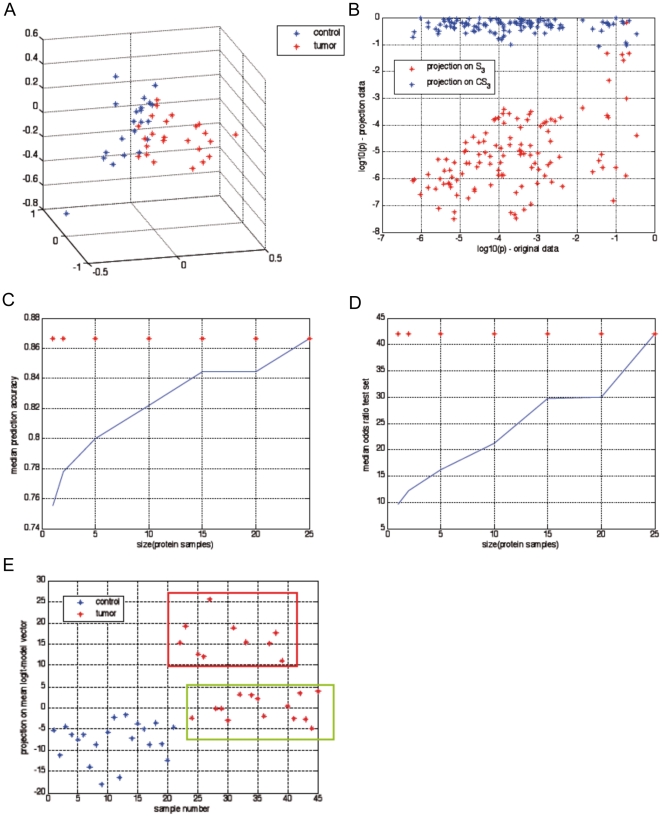
Principal component analysis can separate normal and tumor tissue. (**A**) Scatterplot of the first three principal components of PCA from the protein expression data. The blue stars represent the normal tissues, whereas the red stars show tumors. (**B**) Distribution of information with respect to differential expression between tumor and normal tissues. Each cross represents a protein. The p-values in two-sided t-test are represented by the x-axis, whereas the projections (red: projection onto S3, blue: projection onto complementary space) are represented by the values on the y-axis. Apparently for all proteins with significant differential expression in the original data (log10(p)<−2) the differential expression of the residual component (blue stars) is not significant (log10(p)<−1), whereas the p-values of the PCA-based components (red stars) are similar to the original p-values (x-axis). (**C–D**) Median accuracy and odds ratio of predictive tumor/normal classification. The blue curves show the increase of model quality by increased sample size used for biomarker model. The red stars show the qualities of the logit model based on the first three principal components. (**E**) The output of the regression model (y-axis) indicates the existence of two tumor classes differing significantly according to their separability. Normal tissues (blue and green boxes) using protein expression.

A 3-dimensional scatterplot of the first three principal components of the tissue samples shows a good separation between tumors and normal tissues (Figure 3A). Moreover, figure 3B, depicting the logarithmic p-values of the original data x_i_ for each protein on the x-axis and the components x_p,i_ and x_r,i_ on the y-axis, shows that almost all differential expression can be reflected by the PCA-based component (red stars).

Apparently the differential protein expression between tumors and normal tissues cannot be assigned to one individual protein, but appears to depend on overall protein expression patterns which are represented by only three components of the PCA, in accordance to the results described by Lukk et al. [17]. Hence all (generic, non-redundant) combinations of at least three proteins which can be measured with high accuracy should be sufficient to establish biomarkers with similar predictive performance, if the proteins show a significant contribution to the first three principal components of the PCA. The respective protein set can be selected according to experimental criteria, non-redundancy and significance of correlation to the respective PCA components. Because of the construction of the principal components of PCA as weighted means of the expressions of almost all proteins, the principal components can fill the role of an “intrinsic noise filter”. Hence it is appropriate to express each principal component by the average expression of a (small) group of proteins in order to benefit from the intrinsic noise suppression of averaging as well. Figures 3C–D shows the effect of averaging. The expression of each principal component has been represented by the average expression of 1–25 proteins, which have been randomly selected from the set of 38 proteins with highest correlation to each principal component. Apparently the accuracy and odds ratio depends on the sample size of the proteins used for representation of the principal components. The median of the model qualities is in the same size of the qualities of markers which are based on a biological rationale, indicating that the differential expression may be dominated by large scale, strongly co-regulated protein expression shifts due to tumor formation.

**Figure 4:**
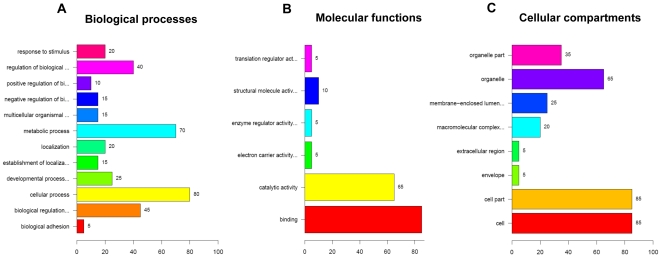
Functional classification of differential expressed proteins in different tumor groups. (**A**) The biological processes, (**B**) molecular functions and (**C**) cellular compartments regulated by the differentially expressed proteins between both tumor groups assessed by Gene Ontology search.

In order to check the expected predictive performance, a logit-model has been identified using only the first three principal components of PCA. In cross-validation (leave-one out) the accuracy of prediction of tumor and normal tissues in the test-set was 86%. Three (of 21) normal tissues and 3 (of 24) tumor tissues have been misclassified. The model shows that the tumors can be split into two groups differing significantly with respect to their separability from normal tissues (Figure 3E and Table 2). Statistical tests showed no direct relation of the tumor groups to the annotated tumor characterizations (Gleason score, PSA marker etc.). Hence either impact factors aside from protein expression may contribute to prostate tumor characteristics, or highly complex, non-monotonic cooperative processes between the proteins have an impact on tumor status which is not covered by the principal components of PCA used in the logit regression model. Functional classification of the 26 identified proteins differentially expressed between both tumor groups are summarized in Figure 4A–C. A total of 70% of the proteins from this dataset were classified to metabolic processes and more than 60% were classified to catalytic or binding functions.

**Table 2:** Proteins with significant differential expression between tumor group 1 and tumor group 2 based on PCA.

S.No	Gene name	Protein name	log10(p), 2-sided t-test
1	EIF4A3	Eukaryotic initiation factor 4A-III	−9,7068
2	RPSA	40S ribosomal protein SA	−8,2847
3	ACTG1	Actin, cytoplasmic 2	−8,2063
4	PPP1CA	Serine/threonine-protein phosphatase PP1-alpha catalytic subunit	−7,9832
5	HSPA5	78 kDa glucose-regulated protein	−7,8941
6	HSP90B1	Endoplasmin	−7,836
7	EEF1G	Elongation factor 1-gamma	−7,7305
8	Albu	Serum albumin	−7,4314
9	TALDO	Transaldolase	−6,9937
10	CRYL1	Lambda-crystallin homolog	−6,917
11	GSTP1	Glutathione S-transferase P	−6,7989
12	HSP90AB1	Endoplasmin	−6,5476
13	NDRG1	Protein NDRG1	−6,1405
14	ACPP	Prostatic acid phosphatase	−6,0939
15	PDIA3	Protein disulfide-isomerase A3	−5,9345
16	PDIA3	Protein disulfide-isomerase A3	−5,8639
17	DPYSL2	Dihydropyrimidinase-related protein 2	−5,7179
18	PDIA3	Protein disulfide-isomerase A3	−5,6059
19	PSMD	26S proteasome non-ATPase regulatory subunit 14	−5,5825
20	PPA2	Inorganic pyrophosphatase 2, mitochondrial	−5,5641
21	Albu	Serum albumin	−5,5566
22	NDUFS1	NADH-ubiquinone oxidoreductase 75 kDa subunit, mitochondrial	−5,4327
23	C7orf24	Uncharacterized protein C7orf24	−5,2732
24	DDAH1	N(G),N(G)-dimethylarginine dimethylaminohydrolase 1	−5,2715
25	P4HB	Protein disulfide-isomerase	−5,1191
26	PGLS	6-phosphogluconolactonase	−5,0895

### Networks analysis of identified proteins in cancer vs. benign samples

Pathways and networks that involved proteins derived from 2-D DIGE experimentations differentially expressed in PCa were analysed using MetaCore™, web-based integrative software. The network architecture represents connections between the individual proteins (nodes).

**Figure 5:**
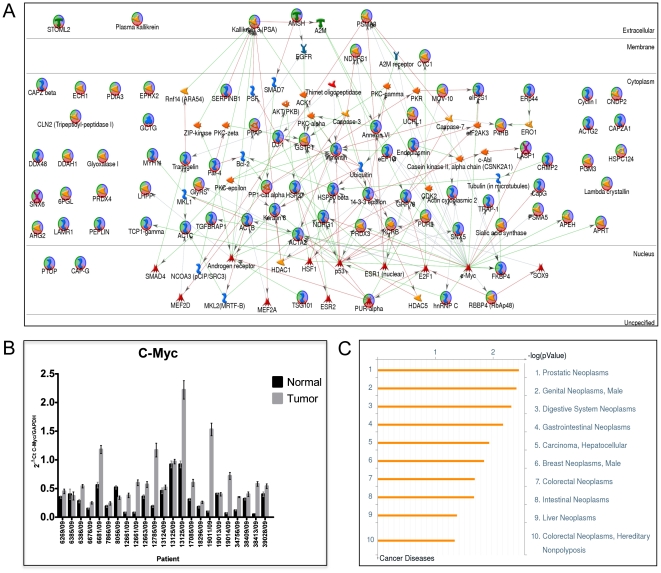
Protein network of differentially expressed proteins in PCa. (**A**) GeneGO MetaCore™ was used to generate a network of direct connections between all identified proteins with altered expression. Red, green, and gray arrows indicate negative, positive, and unspecified effects, respectively. Many of the identified proteins mapped to AR, p53 and c-Myc pathways involved in PCa progression where as some proteins were not connected in network. (**B**) To validate major hubs of the network c-Myc expression at transcriptional level assed by real time PCR from an independent set of samples. Results showed significant increase in the amount of c-Myc mRNA suggests it may have direct/indirect regulation of its connected proteins of shortest network. (**C**) Enrichment of GeneGo diseases by topologically significant proteins identified using all differentially expressed proteins. Data represents differentially expressed proteins mapped to prostatic neoplasms with highest significance followed by male genital neoplasms.

In this analysis hubs (key proteins) of protein networks are proposed to be the key regulatory proteins involved in multicellular processes. The built shortest network (Figure 5A) reveals that c-Myc, p53, androgen receptor, 14-3-3-epsilon, vimentin, PSA and estrogen receptor 1 as network hubs. To validate the network, the highly interconnected hub protein c-Myc mRNA levels measured from independent set of prostate tissues by real-time PCR. Results have confirmed overexpression of c-Myc at mRNA level (Figure 5B) which in turn supports the hypthesis that c-myc protein levels impact on PCa progression as predicted in network. However some of the protein objects were not connected to any of the hubs and shown as unconnected in the network. The proteomic data is analysed for GeneGo biomarker assessment and allows matching the input protein list with known disease profiles consisting of maps, networks and lists of biomarkers known for a disease. Results revealed that the most of the proteins predicted markers for prostate cancer (p = 9.421E-09) as depicted in Figure 5C which supports the current data and provide means to proceed further with validation of unconnected proteins to identify clinically relevant targets for diagnosis and/or prognosis.

The nodes with high degrees of connectivity are considered to be the most important components of a network [18] and due to the high complexity of the network, we analyzed the shortest directed paths using the shortest pathways algorithm and we examined hubs with highest significance.

The functional sub networks were built using MetaCore™ and Ingenuity Pathway Analysis tool from the input proteins as root nodes (Network statistics and Gene Ontology processes are summarized in Table S2. Sub network figures from MetaCore™ analysis were not shown). The most significant sub networks derived from 50 nodes with AR, SRF and TMPRSS2 as network hubs involving tumor suppressor proteins UCHL1, NDRG1 and Par-4. This network may be associated with androgen receptor signalling pathway and cell differentiation. Especially *TMPRSS2* fuses with *ERG* and Ets family genes such as *ETV1*, *ETV4* and *ETV5* in prostate cancers.

**Figure 6:**
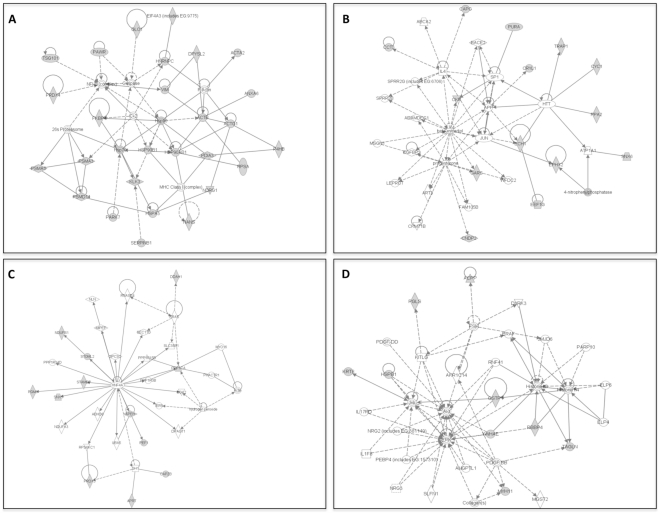
Protein subnetworks of differentially expressed proteins in PCa. (**A–D**) Protein-protein physical/functional interaction sub networks generated by Ingenuity Pathway Analysis tool. Grey filled boxes are the differentially expressed proteins. Only significant sub networks were shown in the figure.

The next significant sub network involves many known proteins to be associated with PCa which may also provide new target proteins which need to be characterized further. This network is probably involved in apoptosis, protein metabolic processes and Ca^2+^ signalling pathways. The sub networks derived from the proteomic data using Ingenuity Pathway Analysis have many common proteins connected with the important hubs such as AR, c-Myc, ERS1, Akt/PKB and their role in PCa progression or potential as disease markers is not known yet (Figure 6A–D). Protein network analysis and clustering of differentially expressed proteins revealed new targets such as DDAH1, ARG2, EIF4A3, Par4, PPA2, Prdx3 and Prdx4, which need further validation to define their potential application in clinical relevance in prostate cancer.

### Confirmation of differentially expressed proteins in PCa

**Figure 7:**
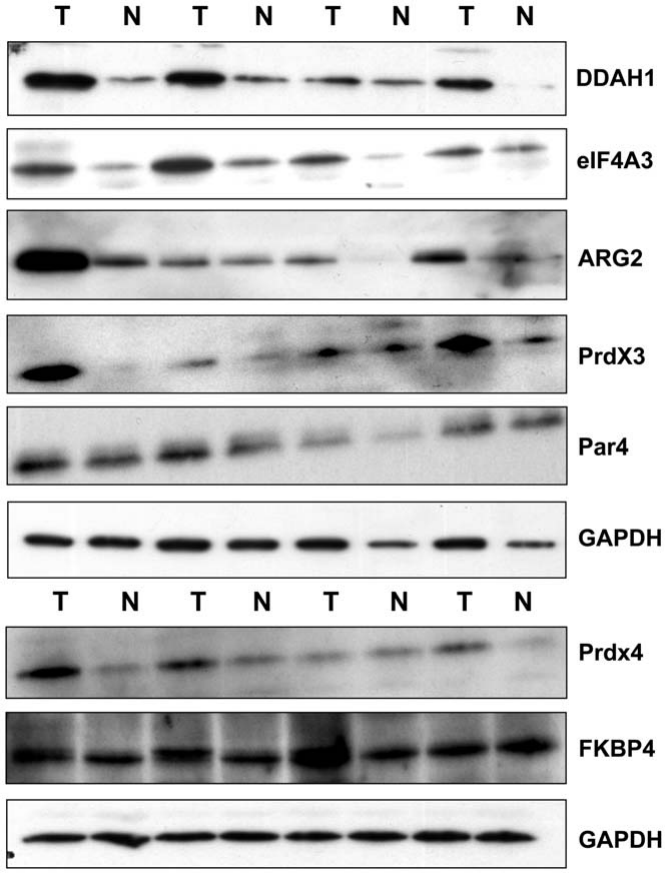
Western blot analysis of DDAH1, ARG2, eIF4A3, Prdx3, Prdx4 and PAWR in benign and PCa tissues. Protein expression identified by western blotting and only representative blots were shown here. The protein expression levels of the analysed target proteins have shown their over expression in PCa compared to normal tissue. GAPDH was used as an internal loading control.

**Figure 8:**
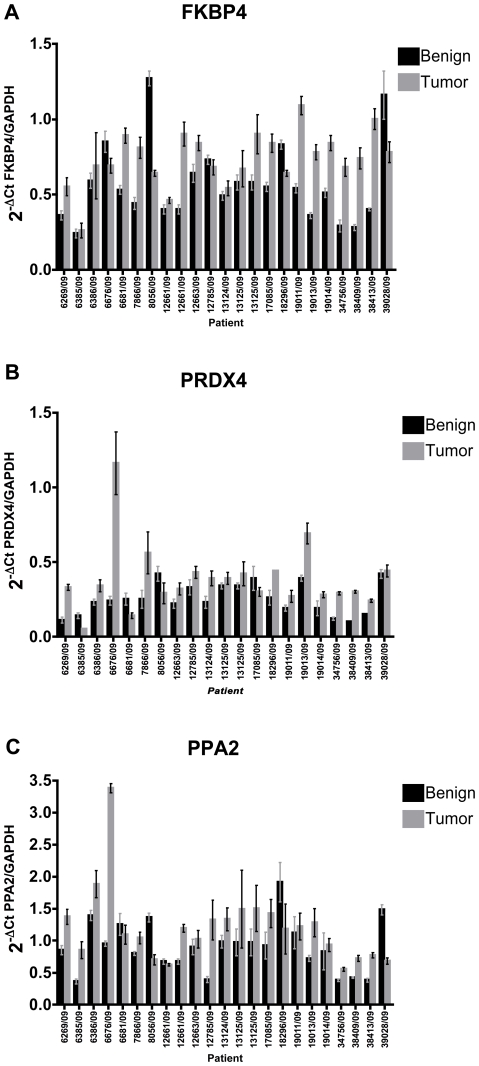
Expression of several protein candidates is regulated at transcriptional level. Quantitative reverse transcription-PCR of transcripts (**A**) FKBP4, (**B**) Prdx4 and (**C**) PPA2 shown from benign prostate tissue (black bars) and localized prostate cancer (grey bars). For statistical significance unpaired student *t*-test performed at 95% CI and p value<0.05 was considered as significant.

In order, to further validate highlighted proteins as described in the previous sections may be useful diagnostic markers and to confirm the 2D-DIGE results and their transcriptional regulation, Western Blot and real-time PCR analysis was performed. The 2-DE protein profile of DDAH1, ARG2, eIF4A3, PPA2, Par-4, Prdx3 and 4 reveals increase in their abundance in PCa patients. Validation of these proteins by Western blotting confirmed significant dysregulation of eIF4A3, ARG2, DDAH1, Par4, Prdx3 and 4 in 79%, 70.1%, 75%, 50.5%, 79%, 70% respectively of tumors compared to corresponding benign tissues included in proteomic study; only one representative blot was shown in Figure 7. The real time PCR results showed significant increase of amount of mRNA for PPA2, Prdx4 and FKBP4 (Figure 8A–C). For DDAH1, ARGI2, eIF4A3, PRDX3 and Par4 results have not shown significant changes in their respective mRNA levels in tumor compared to benign tissues (Data not shown). Taken together, this study confirmed the differential regulation of some of the novel proteins at transcription and/or translational levels in PCa.

## Discussion

The low sensitivity and specificity of current diagnostic methods for prostate cancer underscores the need for improvement in this area. Histological investigation is usually performed on multiple biopsies to distinguish between benign prostate hyperplasia and prostate cancer. In this study, we focused on proteomic analysis of pathological characterized tissue specimens to identify new biomarkers which distinguish PCa from benign prostate tissue. Previously, conventional 2D gel based proteomic studies on PCa identified a large number of differentially expressed proteins and some were reported as potential markers for diagnosis of localized PCa [11], [19]–[22] but none of these markers have yet been introduced into clinical practice. As a limitation, many of the previous studies have been carried to investigate protein expression patterns between tumor and benign tissue from healthy controls. Here, we collected cancer and benign tissues from the same individual prostate gland by manual dissection of frozen tissue for proteome profiling to avoid inter individual differential expression of proteins. Moreover, compared to conventional 2DE, DIGE-based proteomics with fluorescence labelling has many advantages such as higher sensitivity, reproducibility with linear dynamic range for better quantitation, and less technical variations because of a pooled control as internal standard. In the current study, with the ascendancy of manual micro dissection of the prostatectomy specimens and 2D-DIGE gel-based proteomics we focussed to identify novel clinically relevant proteins in PCa.

Our proteomic data on prostate material showed differential expression of 79 proteins in cancer compared to benign tissue. Gene Ontology (GO) search for biological processes classified these proteins to HSP family proteins, signal transmitting proteins, metabolic enzymes, tumor associated proteins, cytoskeletal and oxidative stress controlling proteins involved in tumor progression and dissemination. The list includes many proteins known to be differentially expressed in PCa. As a proof of principle, we found PAPP, a known marker protein for prostate cancer [23] to be up regulated in all PCa samples included in this study. The clustering of samples based on protein expression data did not lead to clusters separating benign from tumor specimen but rather identified a subpopulation of samples that formed a unique cluster. Further data analysis with partition algorithms to find a protein signature which can classify all samples with high certainty revealed more than one protein required in order to define sample identity. Among them, PPA2 is in line with a recent report on its potential as a marker for metastasis of PCa [24]. In order to analyze the impact of co-regulation of protein expression on biomarker identification, unsupervised principal component analysis (PCA) has been applied on the protein expression data (normalized on logarithmic scale), both from tumor and normal tissue samples. Here, in contrast to the cluster analysis we could detect a clear separation of tumor from normal tissue and a separation of two distinct tumor groups based on the individual protein expression patterns found.

In contrast to the cluster analysis, which aims to identify protein groups which show high co-regulation inside a group, but no co-regulation between different groups, PCA is focussed on the representation of the overall variations in the data without any grouping of proteins. Hence, PCA gives good performance in classification of phenotypes with high degree of co-regulated differential expression including various functionalities, in contrast to cluster analysis which is superior if only a few pathways or functionalities are differentially regulated between two phenotypes. Because of these complementary features, a combined approach as presented here can optimize the information retrieval from the data.

In order to put our proteomics data in a biological context, we used a systems biology approach (the platform of specific disease related proteins networks based on the available data bases) as a rational strategy for the identification of novel specific markers and new therapeutic targets. The applicability of the systems biology platform as an approach to biomarker discovery is supported by several observations [25]. The data obtained with 2D-DIGE from prostate tissues was uploaded into MetaCore™ pathways analysis software. MetaCore™ generates an interaction network among the identified proteins and the rest of the protein objects showing physical or functional interactions (e.g., inhibition, activation, modification). The complex network data demonstrates a high number of interactions between differentially expressed proteins and various signalling proteins. Interestingly, there are 4 central hubs including c-Myc, p53, AR and PSA in the network with multiple connections to the rest of the network. These hubs are known to be involved in key cellular processes or have been identified as potential targets in PCa [26]–[30]. To validate the resulting network, our real time PCR results confirmed c-Myc over expression in all samples of this study (Figure 4B). Therefore, c-Myc may be involved in dysregulation of the identified proteins in 2D-DIGE coupled mass spectrometry. Further assessment of the network for biomarker discovery and its assignment to disease entities in MetaCore™ platform significantly scored for prostate cancer. These prediction results together confirmed that the established differentially expressed proteome profile matches with the available database.

When complete proteomic data is analysed for pathway mining the resulting network is highly complex. This led to difficulties in choosing candidate proteins for further validation experiments. Therefore, we built functional sub networks from the significant hubs of main network using IPA software. These sub networks revealed new proteins of unknown function in PCa to be validated as potential biomarkers. However, their precise role in tumor genesis needs to be investigated.

In our study the pathway analysis performed with proteomic data has been interpreted by a theoretical approach that is based on a predetermined database. However, despite its limitations, we strongly believe that this rational strategy will help us to identify candidate biomarkers and proteins that will eventually be validated further as a potential drug targets for prostate cancer.

From pathway analysis, we selected potential candidate proteins for further confirmation of their differential expression. Consistent with our 2D-DIGE proteomic data, the validation by western results demonstrated overexpression of eIF4A3, DDAH1, ARG2, Prdx3, and Prdx4 in significantly high percentage of PCa tissues compared to corresponding benign samples (% of tumors showing differential expression of each protein described in results section).

Eukaryotic initiation factor 4A-III (eIF4A3) is a member of the DEAD box protein family implicated to be involved in various cellular processes such as translation initiation, splicing, ribosome assembly and mRNA export [31]. Based on cDNA microarrays differential expression of eIF4A3 in gastric cancer tissues has been reported [32].

As our data showed over expression of ARG2 in PCa, it is important to note that the protein is also altered in other cancer tissues such as small cell lung cancer where its expression is correlated with the dissemination of cancer cells [33]. ARG2 is known to be involved in polyamine metabolism and polyamines such as ornithine, spermine and spermidine play a critical role in prostate cancer development [34]. ARG2 catalyses the conversion of arginine to ornithine, which is a precursor for the synthesis of polyamines that control growth of benign and tumor cells of the prostate [35]. Previous studies have shown the expression of ARG2 in prostate cancer cell lines and its functional role in prostate cancer development [36], [37].

Two peroxiredoxins such as Prdx3 and Prdx4 are upregulated in PCa determined by expression analysis. Peroxiredoxins are a family of multifunctional antioxidant thioredoxin-dependent peroxidases which have been identified as being differentially expressed in a variety of neoplasms [38]. Oxidative stress by excess production of ROS is involved in activation of signal transduction pathways that are associated with cancer progression [39], [40] [41]. In addition to their role as antioxidative agents, peroxiredoxins are also involved in multi cellular processes such as cell proliferation, apoptosis and gene expression [42]. A previous report described that both Prdx3 and 4 have been associated with the presence of hormone receptors in breast cancer patients [43]. Since prostate tumors are also hormone dependent, we speculate on a role of Prdx3 and 4 controlling tumor proliferation, apoptosis and dissemination of tumor cells during prostate cancer progression. FKBP4 is a peptidyl-prolyl cis-trans-isomerase significantly overexpressed in PCa. Earlier reports described its precise role in tumor initiation and progression via translocation of p53 to the nucleus, leads to p53 inactivation [44]. It is also known as partner involved in AR signalling described as therapeutic target for PCa treatment [45].

Dimethylarginine Dimethyl AminoHydrolase 1 (DDAH1) is known to be involved in NO signalling in cardiovascular disease and pulmonary hypertension. DDAH1 metabolizes dimethyl arginines which act as endogenous inhibitors of nitric oxide synthase (NOS). A recent report suggesting that the intracellular NO promotes androgen independent growth of prostate cancer cells highlights its potential contribution for cancer progression [46]. Moreover, our functional sub network showed connectivity between p53 and the NOS pathway involving DDAH1. However, non-enzymatic activities of DDAH1 are not yet known.

In conclusion, the current study identified potential novel biomarkers for prostate cancer development and/or progression such as eIF4A3, DDAH1, ARG2, Prdx3, and Prdx4 from proteomic data using systems biology approach. Functional validation of these targets will further substantiate their role in the pathophysiology of prostate carcinogenesis and/or as therapeutic targets. Prospective clinical studies will have to confirm their contribution to clinical prostate cancer management as potential prognostic and or predictive biomarkers.

## Supporting Information

Table S1 **Characterisation of differentially expressed proteins.** Identification of 95 differentially expressed protein spots from tumor samples by mass spectrometry using MALDI-TOF-MS/MS. Database IPI human v3.12; cut off score >56 with p-value<0.05, search parameters: MS/MS ion search, enzyme: trypsin, variable modifications: carbamidomethyl (C), oxidation (M), peptide mass tolerance: ±50 ppm, fragment mass tolerance: ±0.45 Da, max missed cleavages: 1.
(DOC)

Table S2 **Statistics of MetaCore™ network analysis of proteomic data and significant functional protein sub networks altered in PCa.** *a* Highly connected sub set of 2-DE identified proteins and key domains of the network. *b* Gene Ontology explain functional processes associated with built network. *c* zScore in MetaCore™ analysis indicates association among the functional sub networks of the differentially expressed protein in 2-DE.
(DOC)
