# Supplementary material for: Identification of Clinically Relevant Protein Targets in Prostate Cancer with 2D-DIGE Coupled Mass Spectrometry and Systems Biology Network Platform
Source: PLoS One. 2011 Feb 11;6(2):e16833. doi: 10.1371/journal.pone.0016833 (PMC3037937; doi:10.1371/journal.pone.0016833)
Supplement: Table S1 — Characterisation of differentially expressed proteins. Identification of 95 differentially expressed protein spots from tumor samples by mass spectrometry using MALDI-TOF-MS/MS. Database IPI human v3.12; cut off score >56 with p-value<0.05, search parameters: MS/MS ion search, enzyme: trypsin, variable modifications: carbamidomethyl (C), oxidation (M), peptide mass tolerance: ±50 ppm, fragment mass tolerance: ±0.45 Da, max missed cleavages: 1. (DOC) [file pone.0016833.s001.doc]

| **S.No** | **Protein name** | **Gene name** | **Hit mass** | **pI** | **No. Of sequenced peptides** | **Hit score** | **Sequence coverage** | **Protein function/Biological processes involved** |
| --- | --- | --- | --- | --- | --- | --- | --- | --- |
| 1 | Adenine phosphoribosyltransferase | APRT | 19595 | 5.82 | 3 | 311 | 80% | Cellular metabolic process |
| 2 | Adenine phosphoribosyltransferase | APRT | 19595 | 5.82 | 1 | 133 | 76% | Cellular metabolic process |
| 3 | Glutathione S-transferase P | GSTP1 | 23341 | 5.30 | 1 | 173 | 68% | Prostate cancer, Glutathione metabolism and anti-apoptosis |
| 4 | Thioredoxin-dependent peroxide reductase, mitochondrial | PRDX3 | 27675 | 7.88 | 3 | 270 | 45% | regulation of metabolic process, activation of NF-kappaB transcription factor |
| 5 | Prostate-specific antigen | KLK3 | 28723 | 7.73 | 1 | 58 | 29% | Prostate cancer/proteolysis |
| 6 | *Protein DJ-1* | *PARK7* | *19878* | *6.78* | *0* | *61* | 65% | Parkinson's disease/neurotransmitter uptake |
| 7 | Heat shock protein beta-1 | HSPB1 | 22768 | 6.36 | 3 | 298 | 61% | Response to unfolded protein/apoptosis |
| 8 | Transgelin | TAGLN | 22596 | 9.29 | 1 | 139 | 74% | Cellular processes |
| 9 | *Phospholysine phosphohistidine inorganic pyrophosphate phosphatase* | *LHPP* | *29147* | *6.04* | *0* | *82* | 64% | Metabolic processes |
| 10 | Delta(3,5)-Delta(2,4)-dienoyl-CoA isomerase, mitochondrial | ECH1 | 35793 | 8.12 | 2 | 282 | 60% | Fatty acid metabolic process |
| 11 | Myosin regulatory light polypeptide 9 | MYL9 | 19814 |  | 2 | 283 | 64% | Regulation of muscle contraction/Cell communication |
| 12 | Uncharacterized protein C7orf24 | C7orf24 | 20994 | 4.79 | 3 | 234 | 70% | Unknown function |
| 13 | Lactoylglutathione lyase | GLO1 | 20764 | 4.92 | 1 | 147 | 40% | Regulation of apoptosis |
| 14 | Proteasome subunit alpha type-3 | PSMA3 | 28415 | 4.97 | 1 | 84 | 35% | Proteasome/ubiquitin-dependent protein catabolic process |
| 15 | Proteasome subunit alpha type-5 | PSMA5 | 26394 | 4.45 | 5 | 510 | 67% | Proteasome/ubiquitin-dependent protein catabolic process |
| 16 | 14-3-3 protein epsilon | YWHAE | 29155 | 4.36 | 2 | 274 | 71% | Signal transduction |
| 17 | Peflin | PEF1 | 30361 | 6.50 | 3 | 141 | 32% | Metabolism |
| 18 | Cytochrome c1, heme protein, mitochondrial | CYC1 | 35367 | 9.25 | 2 | 227 | 28% | Electron transport |
| 19 | 40S ribosomal protein SA | RPSA | 32833 | 4.51 | 5 | 491 | 46% | Ribosome/Translation |
| 20 | Actin, aortic smooth muscle | ACTA2 | 41982 | 5.05 | 4 | 469 | 60% | Vascular smooth muscle contraction |
| 21 | Actin, cytoplasmic 1 | ACTB | 41710 | 5.15 | 5 | 591 | 76% | Cytoskeletal protein/Cell motility |
| 22 | Actin, cytoplasmic 2 | ACTG1 | 41766 | 5.16 | 5 | 578 | 71% | Cytoskeletal protein/Cell motility |
| 23 | Endoplasmin | HSP90B1 | 92411 | 4.48 | 4 | 364 | 41% | Prostate cancer, Cellular homeostasis and Apoptosis |
| 24 | Endoplasmin | HSP90B1 | 92411 | 4.48 | 4 | 276 | 35% | Prostate cancer, Cellular homeostasis and Apoptosis |
| 25 | Putative heat shock protein HSP 90-beta-3 | HSP90AB3P | 68282 |  | 1 | 57 | 19% | Chaperone function/Prostate cancer pathways |
| 26 | Heat shock protein HSP 90-beta | HSP90AB1 | 83212 | 4.68 | 3 | 192 | 39% | Chaperone function/Prostate cancer pathways |
| 27 | Heat shock protein HSP 90-beta | HSP90AB1 | 83212 | 4.68 | 5 | 255 | 38% | Chaperone function/Prostate cancer pathways |
| 28 | 78 kDa glucose-regulated protein | HSPA5 | 72288 | 4.80 | 5 | 724 | 52% | Prion diseases, Antigen processing and presentation |
| 29 | Protein disulfide-isomerase | P4HB | 57081 | 4.49 | 4 | 297 | 45% | Metabolism/protein modification process |
| 30 | Histone-binding protein RBBP4 | RBBP4 | 47626 | 4.50 | 1 | 172 | 25% | DNA binding protein involved in DNA replication |
| 31 | Vimentin | VIM | 53619 | 4.77 | 5 | 517 | 70% | Cell motility/Cellular processes |
| 32 | Vimentin | VIM | 53619 | 4.77 | 5 | 414 | 72% | Cell motility/Cellular processes |
| 33 | Vimentin | VIM | 53619 | 4.77 | 4 | 571 | 77% | Cell motility/Cellular processes |
| 34 | Vimentin | VIM | 53619 | 4.77 | 4 | 563 | 79% | Cell motility/Cellular processes |
| 35 | Vimentin | VIM | 53619 | 4.77 | 5 | 489 | 78% | Cell motility/Cellular processes |
| 36 | Thioredoxin domain-containing protein 4 | TXNDC4 | 46941 | 4.90 | 2 | 310 | 55% | Oxidative metabolism |
| 37 | Heterogeneous nuclear ribonucleoproteins C1/C2 | HNRNPC | 33650 | 4.70 | 1 | 76 | 29% | Nucleic acid metabolism |
| 38 | Peroxiredoxin-4 | PRDX4 | 30521 | 6.24 | 5 | 390 | 35% | Intracellular signaling cascade |
| 39 | 6-phosphogluconolactonase | PGLS | 27530 | 5.95 | 5 | 432 | 60% | Cellular metabolism |
| 40 | F-actin-capping protein subunit beta | CAPZB | 31331 | 5.23 | 1 | 172 | 54% | Cytoskeleton organization and biogenesis |
| 41 | N(G),N(G)-dimethylarginine dimethylaminohydrolase 1 | DDAH1 | 31102 | 5.61 | 2 | 299 | 61% | Nitric oxide mediated signal transduction |
| 42 | Stomatin-like protein 2 | STOML2 | 38510 | 7.50 | 2 | 181 | 68% | Unknown function |
| 43 | PRKC apoptosis WT1 regulator protein | PAWR | 36545 | 5.05 | 1 | 81 | 13% | Regulation of Apoptosis |
| 44 | Protein NDRG1 | NDRG1 | 42808 | 5.67 | 3 | 197 | 45% | Cell differentiation |
| 45 | Mannose-6-phosphate isomerase | MPI | 46626 |  | 3 | 256 | 45% | Carbohydrate Metabolism |
| 46 | Arginase-2, mitochondrial | ARG2 | 38554 | 6.45 | 3 | 402 | 53% | Nitrogen compound metabolic process |
| 47 | Creatine kinase B-type | CKB | 42617 | 5.30 | 1 | 55 | 27% | Unknown function |
| 48 | Creatine kinase B-type | CKB | 42617 | 5.30 | 1 | 136 | 40% | Unknown function |
| 49 | Lambda-crystallin homolog | CRYL1 | 35396 | 6.10 | 3 | 236 | 50% | Lipid metabolic process |
| 50 | Serine/threonine-protein phosphatase PP1-alpha catalytic subunit | PPP1CA | 37488 | 6.25 | 3 | 266 | 57% | Insulin signaling pathway, Focal adhesion, Cell cycle |
| 51 | 26S proteasome non-ATPase regulatory subunit 14 | PSMD14 | 34555 | 6.51 | 2 | 153 | 51% | Proteasome, protein folding and sorting |
| 52 | Inorganic pyrophosphatase 2, mitochondrial | PPA2 | 37896 | 7.45 | 2 | 119 | 35% | Cellular metabolic process |
| 53 | Delta(3,5)-Delta(2,4)-dienoyl-CoA isomerase, mitochondrial | ECH1 | 35793 | 8.12 | 3 | 395 | 64% | Unknown function |
| 54 | Transcriptional activator protein Pur-alpha | PURA | 34889 | 6.39 | 3 | 346 | 43% | Cell cycle, regulation of transcription |
| 55 | Leukocyte elastase inhibitor | SERPINB1 | 42715 | 6.19 | 4 | 323 | 50% | Unknown function |
| 56 | Prostatic acid phosphatase | ACPP | 44537 | 6.19 | 2 | 146 | 35% | Prostate cancer, Proteolysis |
| 57 | Macrophage-capping protein | CAPG | 38494 | 6.18 | 3 | 308 | 37% | Actin cytoskeleton organization and biogenesis |
| 58 | Myosin-11 | MYH11 | 227199 | 5.18 | 2 | 111 | 15% | Actin cytoskeleton organization and biogenesis |
| 59 | Sialic acid synthase | NANS | 40281 | 6.73 | 4 | 460 | 67% | carbohydrate and lipid metabolic process |
| 60 | Keratin, type II cytoskeletal 8 | KRT8 | 53671 | 5.26 | 4 | 306 | 56% | Cytoskeletal protein |
| 61 | Uncharacterized protein C20orf166 | C20orf166 | 12402 | 6.88 | 1 | 57 | 69% | Un known function |
| 62 | Keratin, type II cytoskeletal 8 | KRT8 | 53671 | 5.26 | 4 | 414 | 57% | Cytoskeletal protein |
| 63 | Protein disulfide-isomerase A3 | PDIA3 | 56747 | 6.28 | 3 | 510 | 60% | Apoptosis, cell redox homeostasis |
| 64 | Protein disulfide-isomerase A3 | PDIA3 | 56747 | 6.28 | 3 | 494 | 60% | Apoptosis, cell redox homeostasis |
| 65 | Cytosolic non-specific dipeptidase | CNDP2 | 52845 | 5.81 | 2 | 267 | 59% | Proteolysis |
| 66 | Protein disulfide-isomerase A3 | PDIA3 | 56747 | 6.28 | 4 | 436 | 53% | Apoptosis, cell redox homeostasis |
| 67 | Sorting nexin-6 | SNX6 | 46620 | 6.05 | 1 | 207 | 42% | Transcription, protein transport |
| 68 | Tumor susceptibility gene 101 protein | TSG101 | 43916 | 6.39 | 3 | 150 | 41% | Ubiquitin cycle, endocytosis |
| 69 | Serum albumin | Albu | 69321 | 6.21 | 3 | 225 | 45% | ------ |
| 70 | STAM-binding protein | STAMBP | 48047 | 6.24 | 1 | 103 | 37% | Ubiquitin cycle |
| 71 | Serum albumin | Albu | 69321 | 6.21 | 2 | 63 | 30% | ------ |
| 72 | Serum albumin | Albu | 69321 | 6.21 | 2 | 109 | 23% | ------ |
| 73 | Sorting nexin-5 | SNX5 | 46787 | 6.75 | 1 | 122 | 30% | Transcription, protein transport |
| 74 | Elongation factor 1-gamma | EEF1G | 50087 | 6.64 | 3 | 299 | 42% | Translational elongation |
| 75 | Eukaryotic initiation factor 4A-III | EIF4A3 | 46841 | 6.69 | 2 | 353 | 68% | Transcriptional regulation |
| 76 | Phosphoacetylglucosamine mutase | PGM3 | 59814 | 6.21 | 1 | 92 | 29% | Carbohydrate Metabolism |
| 77 | Epoxide hydrolase 2 | EPHX2 | 62575 | 6.21 | 4 | 293 | 33% | Lipid Metabolism |
| 78 | T-complex protein 1 subunit gamma | CCT3 | 60495 | 6.44 | 2 | 190 | 53% | Protein folding |
| 79 | Dihydropyrimidinase-related protein 2 | DPYSL2 | 62255 | 6.33 | 5 | 543 | 60% | Cell differentiation |
| 80 | Heat shock protein 75 kDa, mitochondrial | TRAP1 | 80060 | 8.40 | 3 | 220 | 41% | Cell differentiation |
| 81 | Heat shock protein 75 kDa, mitochondrial | TRAP1 | 80060 | 8.40 | 3 | 329 | 50% | Cell differentiation |
| 82 | Glycyl-tRNA synthetase | GARS | 83087 | 7.04 | 0 | 88 | 36% | Translation |
| 83 | Annexin A6 | ANXA6 | 75826 | 5.28 | 2 | 322 | 63% | Metabolism |
| 84 | NADH-ubiquinone oxidoreductase 75 kDa subunit, mitochondrial | NDUFS1 | 79417 | 6.11 | 4 | 247 | 42% | Electron transport |
| 85 | Acylamino-acid-releasing enzyme | APEH | 81173 | 5.16 | 1 | 226 | 34% | Proteolysis |
| 86 | Eukaryotic translation initiation factor 2 subunit 1 | EIF2S1 | 36089 | 4.73 | 3 | 292 | 66% | Regulation of translation |
| 87 | Ubiquitin carboxyl-terminal hydrolase isozyme L1 | UCHL1 | 24808 | 5.18 | 0 | 71 | 65% | Ubiquitin-dependent protein catabolic process |
| 88 | LIM and SH3 domain protein 1 | LASP1 | 29698 | 7.07 | 3 | 191 | 37% | Cytoskeleton organization and biogenesis |
| 89 | 2-oxoisovalerate dehydrogenase subunit alpha, mitochondrial | ODBA | 50439 | 8.42 | 12 | 128 | 31% | Branched chain family amino acid metabolic process |
| 90 | Alpha-enolase | ENO1 | 47139 | 7.01 | 17 | 134 | 55% | Fatty acid metabolism |
| 91 | Lipoamide acyltransferase component of branched-chain alpha-keto acid dehydrogenase complex, mitochondrial | ODB2 | 53453 | 8.71 | 24 | 289 | 42% | Metabolism |
| 92 | Tripeptidyl-peptidase 1 | TPP1 | 61210 | 6.01 | 5 | 197 | 56% | Proteolysis |
| 93 | Serine/threonine-protein phosphatase PP1-alpha catalytic subunit | PPP1CA | 37488 | 5.94 | 20 | 241 | 57% | Unknown function |
| 94 | F-actin-capping protein subunit alpha-1 | CAPZA1 | 32902 | 5.42 | 2 | 287 | 72% | Metabolism |
| 95 | FK506-binding protein 4 | FKBP4 | 51641 | 4.97 | 3 | 209 | 48% | Signal transduction |
| 96 | FK506-binding protein 4 | FKBP4 | 51641 | 4.97 | 3 | 215 | 47% | Signal transduction |
