# Supplementary material for: Identification of Clinically Relevant Protein Targets in Prostate Cancer with 2D-DIGE Coupled Mass Spectrometry and Systems Biology Network Platform
Source: PLoS One. 2011 Feb 11;6(2):e16833. doi: 10.1371/journal.pone.0016833 (PMC3037937; doi:10.1371/journal.pone.0016833)
Supplement: Table S2 — Statistics of MetaCore™ network analysis of proteomic data and significant functional protein sub networks altered in PCa. a Highly connected sub set of 2-DE identified proteins and key domains of the network. b Gene Ontology explain functional processes associated with built network. c zScore in MetaCore™ analysis indicates association among the functional sub networks of the differentially expressed protein in 2-DE. (DOC) [file pone.0016833.s002.doc]

| **S. No** | **Network** | **GO Processes** | **Root nodes** | **p-Value** | **zScore** |
| --- | --- | --- | --- | --- | --- |
| 1 | DJ-1, TSG101, ACTC, Transgelin, ACTA2 | cell differentiation, cellular developmental process, androgen receptor signaling pathway | 16 | 2.060E-35 | 63.89 |
| 2 | RBBP4 (RbAp48), DDX48, APRT, eEF1G, Actin cytoplasmic 2 | regulation of mitochondrial membrane potential, calcium-dependent cell-matrix adhesion, translational elongation | 15 | 6.550E-33 | 60.58 |
| 3 | Endoplasmin, GRP78, eIF2S1, PDIA3, Annexin VI | regulation of Apoptosis, regulation of cell death, regulation of developmental process, posttranscriptional regulation of gene expression | 11 | 7.930E-22 | 41.1 |
| 4 | Plasma kallikrein, hnRNP C, FKBP4, PDIA3, KCRB | developmental growth, response to stress | 11 | 7.930E-22 | 41.1 |
| 5 | DJ-1, KCRB, ACTB, CapG, 14-3-3 epsilon | regulation of Apoptosis, cellular component organization | 10 | 7.030E-20 | 38.95 |
| 6 | Kallikrein 3 (PSA), SERPINB1, Annexin VI, Plasma kallikrein, GRP78 | regulation of cell migration, protein metabolic process, response to external stimulus | 10 | 1.760E-19 | 37.34 |
| 7 | HSP27, TSG101, PP1-cat alpha, Transgelin, ACTA2 | regulation of developmental process, positive regulation of cellular process | 10 | 1.760E-19 | 37.34 |
| 8 | PDIA3, PRDX3, Glyoxalase I, FKBP4, PSMA3 | multi-organism process, cell redox homeostasis | 8 | 1.520E-16 | 36.81 |
| 9 | PUR2, GlyRS, LHPP, SNX5, DDAH1 | ER-nuclear signaling pathway, response to endoplasmic reticulum stress | 7 | 1.780E-13 | 36.43 |
| 10 | AMSH, CapG, CRMP2, GSTP1, Annexin VI | regulation of phosphorus metabolic process, intracellular signaling cascade | 9 | 3.420E-17 | 33.58 |
| 11 | GSTP1, HSP27, PP1-cat alpha, Keratin 8, Par-4 | regulation of cell death, response to chemical stimulus | 8 | 1.570E-15 | 32.19 |
| 12 | CLN2 (Tripeptidyl-peptidase I), DJ-1, UCHL1, GCTG, PTOP | regulation of telomere maintenance | 7 | 7.040E-14 | 30.79 |
| 13 | Vimentin, HSP90 beta, HSP27, Transgelin, ACTG2 | regulation of apoptosis, positive regulation of cellular process | 7 | 1.060E-13 | 29.96 |
| 14 | PUR-alpha, ACTA2, ACTB, LAMR1, Actin cytoplasmic 2 | response to hormone stimulus, response to steroid hormone stimulus | 8 | 5.760E-15 | 29.82 |
| 15 | HSP27, DJ-1, Kallikrein 3 (PSA), GRP78, PSMA3 | regulation of molecular function, regulation of cell proliferation | 6 | 1.010E-10 | 22.3 |
